# Decrease of Clone Diversity in IgM Repertoires of HBV Chronically Infected Individuals With High Level of Viral Replication

**DOI:** 10.3389/fmicb.2020.615669

**Published:** 2021-01-15

**Authors:** Binbin Hong, Lizhi Wang, Chunlan Huang, Xiaoju Hong, Alan Liu, Qiulan Li, Qiaoling Liu, Lili Su, Lixing Wang, Chunyu Wang, Tianlei Ying

**Affiliations:** ^1^Central Laboratory, Second Affiliated Hospital, Fujian Medical University, Quanzhou, China; ^2^Traditional Chinese Medicine Department, Rehabilitation Hospital, Quanzhou, China; ^3^Key Laboratory of Medical Molecular Virology of Ministries of Education and Health, School of Basic Medical Sciences, Fudan University, Shanghai, China

**Keywords:** high-throughput sequencing, antibody repertoire, chronic HBV infection, immunoglobulin M, clone diversity

## Abstract

High-throughput antibody sequencing allows in-depth insights into human antibody repertoires. To investigate the characteristics of antibody repertoires in patients with chronic HBV infection, we performed Illumina sequencing and IMGT/HighV-QUEST analysis of B lymphocytes from healthy adults and the HBV carriers with high or low level of viral replication. The comparative study revealed high levels of similarity between the IgM and IgG repertoires of the HBV carriers and the healthy adults, including the somatic mutations in V regions, the average CDR3 length, and the occurrence of junctional modifications. Nevertheless, the diversity of the unique clones decreased and some clusters of unique clones expanded in the IgM repertoire of chronic HBV carriers (CHB) compared with healthy adults (HH) and inactive HBV carriers (IHB). Such difference in clone diversity and expansion was not observed in the IgG repertoires of the three populations. More shared antibody clones were found between the IgM repertoires of IHB and HH than that found between CHB and HH (7079 clones vs. 2304 clones). Besides, the biased used IGHD genes were IGHD2-2 and IGHD3-3 in CHB library but were IGHD3-10 and IGHD3-22 in IHB and HH library. In contrast, for IgG repertories, the preferred used VDJ genes were similar in all the three populations. These results indicated that low level of serum HBV might not induce significant changes in BCR repertoires, and high level of HBV replication could have more impacts on IgM repertories than IgG repertoires. Taken together, our findings provide a better understanding of the antibody repertoires of HBV chronically infected individuals.

## Introduction

Chronic hepatitis B is a major global health issue. More than 200 million people are persistently infected with the hepatitis B virus (HBV) worldwide, and are at high risk of developing liver cirrhosis and hepatocellular carcinoma (El-Serag, 2012; Trepo et al., 2014). However, current therapies using interferon or direct-acting antiviral agents are unlikely to eradicate the virus or cure the infection (Gish et al., 2012; Revill et al., 2016); thus, efficient therapeutic approaches are urgent requirements.

The virus-host interaction during HBV infection determines infection outcome. Several reviews have summarized the current knowledge of host immune responses in chronic HBV infections (Bertoletti and Ferrari, 2012, 2016; Lin and Kao, 2016; Shin et al., 2016; Gehring and Protzer, 2019). Generally, defective immune responses, particularly the exhaustion of T lymphocytes, were commonly observed during chronic HBV infection. Thus, a series of strategies were designed to restore human immunity and resolve the infection. Recently, several such agents have been developed to reconstitute host responses including lymphotoxin-β receptor agonists, toll-like receptor agonists, immune checkpoint inhibitors, and therapeutic vaccines (Gehring and Protzer, 2019). Notably, the discovery of the sodium taurocholate co-transporting polypeptide (NTCP) as the receptor of HBV has largely advanced our understanding of humoral immune responses and anti-HBV antibodies during chronic HBV infection, which was largely neglected in previous HBV studies (Yan et al., 2012; Ni et al., 2014). The NTCP interacts directly with the preS1 domain of the HBV surface antigen (HBsAg), and antibodies against HBsAg could block the virus entry into cells, neutralize the virus, and restrict the spread of HBV (Wu et al., 2019; Yan et al., 2019). Thus, antibodies play important roles in limiting HBV infections and eliminating the virus.

Recently, the analysis of human antibodies based on high-throughput sequencing and bioinformatics methods have provided unprecedented insight into human immune repertoires. Several studies have revealed the antibody repertoire dynamics before and after HBV vaccination using high-throughput sequencing (Chang et al., 2016; Ma et al., 2017; Miyasaka et al., 2019). However, very few studies investigated the antibody repertoires of individuals with chronic HBV infection, except one study reporting the characteristics of immunoglobulin G (IgG) repertoire of HBV-infected children, which uncovered a network of sequence-related heavy-chain complementarity determining region 3 (CDR3) clones in HBV carriers (Chang et al., 2016). Therefore, the understanding of the characteristics of antibody repertoires with chronic HBV infection remains limited, and whether defects exist in antibody production or function during HBV infection has not yet been fully elucidated.

In this study, we describe the characteristics of B cell repertoires in individuals with chronic HBV infections, and the comparison with that of healthy adults. The heavy chains from immunoglobulin M (IgM) and IgG were analyzed using the Illumina sequencing platform to investigate the influence of HBV infection on the antibody repertoires. Although most of the characteristics were similar among the repertoires of healthy adults and HBV carriers, we found some important features of the IgM repertories in HBV carriers induced by the high level of HBV replication, including the decrease of the clone diversity, the expansion of the clone size and the different biased usage of IGHD and IGHJ genes.

## Materials and Methods

### Samples

Thirty adults were employed in our study. They comprised 10 inactive HBV carriers with serum HBsAg but without serum virus load increased (IHB), 10 chronic HBV carriers with elevated HBV load levels (CHB), and 10 healthy adults (HH) who underwent a routine health check with no history of HBV infection or known major diseases. None of the included HBV carriers had active hepatitis or received antiviral treatment. The basic characteristics of the study population were summarized in Table 1.

**TABLE 1:** The basic characteristics of study population.

Group	Gender (F/M)^*a*^	Average Age^*b*^	Five serological markers of HBV^*c*^					HBV DNA load^*d*^ (IU/mL)
				
			HBsAg	HBsAb	HBeAg	HBeAb	HBcAb	
Chronic HBV carriers	2/8	40.0 ± 10.1	+	−	±	+	+	8.62 × 10^5^
Inactive HBV carriers	2/8	44.3 ± 9.27	+	−	−	+	+	<500^*e*^
Healthy adults	0/10	37.0 ± 9.55	−	−	−	−	−	0

*^a^F, female; M, male. ^b^The average age had no significant difference in the three groups (Student’s t-test, p > 0.05). ^c^Five serological markers of HBV were tested by ELISA. ^d^HBV DNA load was tested by the real-time fluorescent quantitative PCR. ^e^The detection limit is 500 IU/mL.*

### Establishment of the Antibody Repertoires for Deep Sequencing

Total RNA was extracted from peripheral blood mononuclear cells (PBMCs) and prepared according to the reported protocols (Chen et al., 2009), then reverse-transcripted to the first-strand cDNA and used as the source for amplification of antibody sequences. The PCR conditions and primers employed in amplifying the IgM heavy chains repertoire have been previously reported (Hong et al., 2018). The primers used to amplify the variant (V) segments of IgG repertoire were the same as those for the IgM repertoire, but the amplification of the constant domains were performed using a sense primer specific for the CH1 domain of IGHG spanning the first eight codons (3’–5’ strand) according to the ImMunoGeneTics database^1^ (Lefranc et al., 2015). Two rounds of PCR amplification were performed to produce special isotype antibody fragments with the proper length for the Illumina sequencing. PCR amplifications were performed in a 50 μL volume, using 25 μL Pfu master mix (CWbio, China), 1 μL template, and 1 μL (50 nM) of each primer mixture. The PCR conditions were as follows: initial denaturation at 94°C for 5 min, 35 cycles of denaturation at 94°C for 30 s, annealing at 56°C for 1 min, extension at 72°C for 1 min, and final extension at 72°C for 10 min. The PCR amplicons were purified and subjected to high-throughput sequencing based on the Illumina Hiseq platform according to the manufacturer’s protocol.

### Sequence Processing

To exclude biologically implausible sequences, a series of stringent quality control criteria were applied in the data processing. A detailed explanation of these criteria has been published in our previous report (Hong et al., 2018). IMGT/High V-QUEST (version 1.5.1) was used for sequence annotation to determine the V(D)J genes, CDRs, junctional modifications, and to identify insertion and deletion (indels) errors (Ehrenmann et al., 2011; Lefranc, 2011). The data were classified into productive and unproductive sequences according to the analysis of IMGT/HighV-QUEST. The unproductive V(D)J rearrangements were eliminated from the dataset. Then, the productive sequences that contained stop codons, indel errors, and substitutions or mutations in the conserved amino acids at specified positions were excluded. Furthermore, the redundant sequences were eliminated to avoid the accumulation of one single sequence due to PCR amplification. Finally, the sequences that had unique VDJ gene rearrangements, including those contained unique CDR3 amino acid sequences, or had identical CDR3 but distinct VDJ rearrangements were defined as the unique clone. Additionally, the clones with the same V(D)J gene rearrangements and the same CDR3 sequences were assigned to a unique cluster. The number of sequences after each step of processing were listed in Table 2.

**TABLE 2:** The number of input cells and sequencing data.

Library	Healthy human (HH)		Individuals with HBV infection			
			Inactive HBV carriers (IHB)		Chronic HBV carriers (CHB)	
Repertoires	IgM	IgG	IgM	IgG	IgM	IgG
Total input cells	1.0 × 10^7^		1.0 × 10^7^		1.0 × 10^7^	
Raw seqs*	14,179,342	10,560,609	13,755,568	14,615,173	13,670,308	13,486,586
Productive seqs	7,743,758	6,273,616	7,597,821	8,661,966	7,901,357	7,958,448
Unique amino acid seqs	4,527,647	2,799,053	4,439,906	3,526,681	4,412,407	3,479,419
Unique clones	510,607	139,969	544,159	165,050	464,874	176,100

*seqs, sequences.

### Statistical Analyses

The Margalef index (*D*) was used to describe the repertoire diversity, which was defined and given by the function *D* = (S−1)/ln N, with S being species richness and N being the total number of all specimens in a sample (Li et al., 2016). The species richness in our study were the number of the unique clones that extracted from the datasets of unique amino acid sequences. The *D*-values were calculated, respectively, when 10, 100, 1,000, 10,000, 100,000, 1,000,000, 1,500,000 sequences were randomly selected. Moreover, the percentage change was calculated to assess the difference between two *D*-values, and the percentage difference not less than 20% was considered to be significant. Notably, except for the calculation of the repertoire diversity, the unique clones finally obtained from all the sequences were used in the data analysis of the other repertoire characteristics in this study.

Data analyses were performed using the R, Perl, and GraphPad Prism programs. Student’s *t*-test, Pearson’s chi-test, and logistic regression analysis were used to determine the statistical significance when required. The effect sizes were also used in this study to determine whether meaningful differences exist when the *p-*value is small. *Cohen’s d* value was used to measure the standardized difference between two means, and *odds ratios (ORs)* and the 95% confidence interval (95%CI) were used as the effect size measure between two rates (Cohen, 1988; Muth, 2006). In comparative analyses, to simplify these criteria, the difference was considered to be significant when *p* < 0.05 (two sided), *d* ≥ 0.20 and *OR* ≥ 1.50 or ≤ 0.60. The sequencing data have been deposited in the NCBI SRA database (Accession number: PRJNA578020)^2^.

### Ethics Statement

The blood samples were provided by the Second Affiliated Hospital of Fujian Medical University (Quanzhou, Fujian, China) with the approval of the institutional research board and the donors’ consent. Procedures followed in this study were under the ethical standards of concerned institutional policies.

## Results

### The Repertoire Diversity

In this study, we carried out high-throughput sequencing analysis of the BCR repertoires from individuals with chronic HBV infection and compared them with the repertoires from healthy adults (HH). The HBV-infected individuals were divided into two groups according to the level of serum HBV load: chronic HBV carriers with a high level of virus load (CHB) and inactive HBV carriers with no increase of virus load (IHB). Initially, approximately 1 × 10^7^ PBMCs from each investigated group were input into the analysis and yielded more than 1 × 10^8^ raw reads in each library after the sequencing reactions. The sequences that had unique V(D)J gene rearrangements or unique CDR3 amino acid sequences were defined as the unique clone in our study. After a series of stringent data filtering and cleaning procedures, 510,607 unique clones were identified in the IgM repertoire of HH library, 544,159 unique clones in IHB library and 464,874 clones in CHB library. Besides, 139,969 unique clones were found in the IgG repertoire of HH library, 165,050 unique IgG clones in IHB library and 176,100 unique clones in CHB library (Table 2).

**FIGURE 1:**
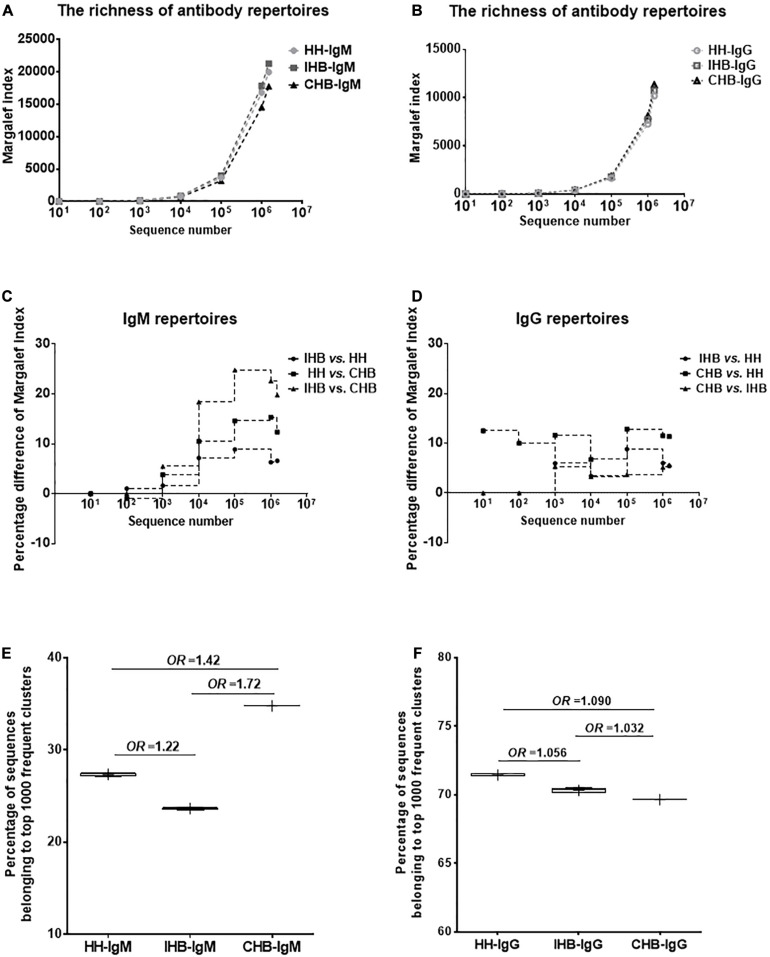
The Margalef index (*D*) was used to describe the repertoire diversity that was calculated, respectively, when 10, 100, 1,000, 10,000, 100,000, 1,000,000 sequences were randomly selected using the randomized table generated by R program. **(A)** The diversity of the unique clones in the IgM repertoires of healthy adults (HH), inactive HBV carriers (IHB) and chronic HBV carriers (CHB). **(B)** The diversity of the unique clones in the IgG repertoires of HH, IHB, and CHB libraries. **(C)** The percentage difference of the *D*-values between each two libraries in IgM repertoires. **(D)** The percentage difference of the *D*-values between each two libraries in IgG repertoires. **(E)** The percentage of the total repertoire comprising by top 1,000 most frequent clusters were selected from the datasets of 100,000 sequences in IgM repertoires of HH, IHB and CHB libraries. **(F)** The percentage of the total repertoire comprising by top 1,000 most frequent clusters were selected from the datasets of 100,000 sequences in IgG repertoires of HH, IHB, and CHB libraries.

To compare the repertoire diversity of the three libraries, the Margalef index (*D*) were calculated based on the same amount of sequence number randomly selected from each dataset (Figures 1A,B). In IgM repertoires, the *D*-values of IHB library were slightly higher than that of HH library, but the percentage difference of the *D*-values were not more than 10%, suggesting that the diversity of unique clones showed no significant difference between HH and IHB library (Figure 1C). In contrast, the *D*-values of CHB library were lower than that of HH and IHB library. The greatest percentage difference of *D*-values between HH and CHB library was 15.33% when 1,000,000 sequences were included in calculations, and the percentage difference decreased to 12.36% as the sequence number increased to 1,500,000. The difference of *D*-values was larger between IHB and CHB library, which was 24.76 and 22.59% when 100,000 and 1,000,000 sequences included, respectively, and the percentage difference decreased to 19.76% when 1,500,000 sequences were included (Figure 1C). However, the *D*-values showed slight difference among the IgG repertoires of HH, IHB and CHB library; the difference of the *D*-values between each two libraries were less than 13% when the included sequence number increased from 10 to 1,500,000 (Figure 1D). Together, these results suggested that the diversity of the unique clones had no significant difference in the IgG repertoires of the three libraries, but the diversity of the unique clones decreased greatly in the IgM repertoire of CHB compared with IHB library when the included sequence number were relatively less.

Since the diversity of the unique clones differed greatly in the IgM repertoires when 100,000 sequences were included, the clonal expansion was analyzed using the datasets of 100,000 sequences. To evaluate the clone expansion, the number of the unique amino acid sequences belonging to each unique clusters were calculated that represented the size of the cluster. Then, the top 1,000 most frequent clusters were selected and the proportions of sequences belonging to these clusters in the total repertoire were calculated. The percentage was 34.76% in CHB, 27.37% in HH and 23.37% in IHB; which suggested that the degree of the clone expansion in the IgM repertoire of CHB library was significantly higher than IHB library (*p* < 2.2E-16, *OR* = 1.720, 95%CI: 1.687–1.754; Figure 1E), and was slightly higher than HH library (*p* < 2.2E-16, *OR* = 1.416, 95%CI: 1.390–1.443; Figure 1E). Interestingly, the degree of the clone expansion did not show significant difference between the IgM repertoire of HH and IHB library (*p* < 2.2E-16, *OR* = 1.215, 95%CI: 1.190–1.239; Figure 1E). Besides, the degree of the clone expansion did not show significant difference between the IgG repertoire of HH, IHB, and CHB library, since the *OR* values were close to 1.0 (Figure 1F).

### The Repertoire Similarity

**FIGURE 2:**
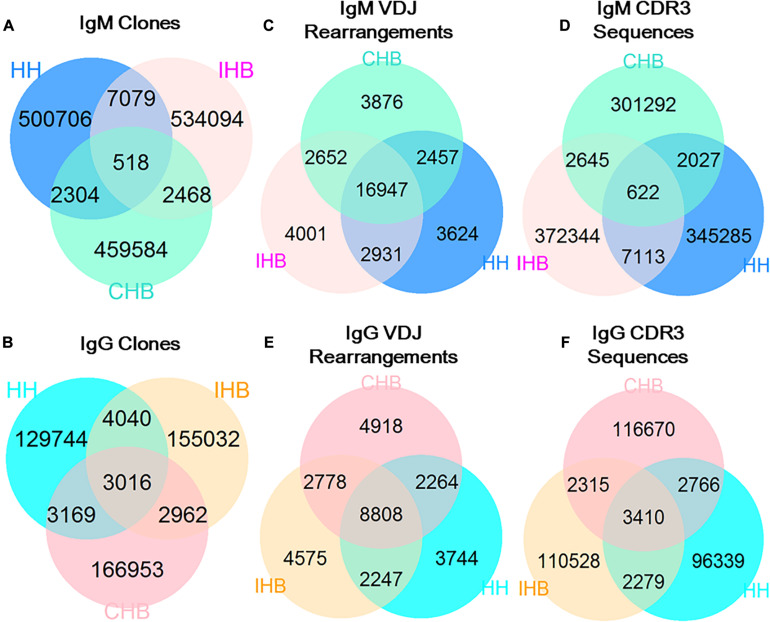
The shared unique antibody clones in HH, IHB and CHB. **(A)** The shared unique clones in IgM repertoires of HH, IHB and CHB. **(B)** The shared unique clones in IgG repertoires. **(C)** The shared V(D)J gene rearrangements in IgM repertoires of HH, IHB and CHB. **(D)** The shared V(D)J gene rearrangements in IgG repertoires. **(E)** The shared CDR3 sequences in IgM repertoires of HH, IHB and CHB. **(F)** The shared CDR3 sequences in IgG repertoires.

To assess the repertoire similarity, we calculated the proportion of the shared clones among the antibody repertoires from the three libraries. In IgM repertoires, only 518 unique clones shared in HH, CHB, and IHB libraries (a collection designated as “ALL_*shared*_”) constituting 0.10% in HH libraries, suggesting most of the clones were different among the three libraries. Besides the clones of ALL_*shared*_, there were 7,079 shared clones found between IHB and HH library (HH + IHB_*shared*_) accounting for 1.30% of IHB, but only 2,304 clones shared between CHB and HH library (HH + CHB_*shared*_), accounting for 0.5% of CHB (Figure 2A). The proportion of clones in HH + IHB_*shared*_ was higher than that in HH + CHB_*shared*_. Although the shared clones account for a small portion of the repertoires, the difference has statistical significance in the comparative analysis based on the large sample size (*p* < 2.2E-16, *OR* = 2.646, 95%CI: 2.524–2.774). In IgG repertoires, there were 3,016 clones in ALL_*shared*_, accounting for 2.15% in HH, of which the proportion was much higher in IgG repertoires than that of IgM repertories (Figure 2B). The proportion of IgG clones in HH + IHB_*shared*_ was 2.45%, while it was 1.80% in HH + CHB_*shared*_; but the difference was slight between the two collections (2.45% vs. 1.80%, *p* < 2.2E-16, OR = 1.371, 95%CI: 1.308–1.437).

Then, the shared portions in unique V(D)J rearrangements and unique CDR3s between the libraries were calculated separately to find the factors contributed to the converging clones among the libraries. The majority of V(D)J rearranged patterns were commonly observed in the IgM repertoires of all the three libraries. The proportion of ALL_*shared*_ was 65.3%, and the proportion of HH + IHB_*shared*_ and HH + CHB_*shared*_ was 11.05 and 9.47%, respectively, indicating that the difference was not significant (*OR* = 1.187, 95%CI: 1.121–1.256; Figure 2C). In contrast, only 0.2% of CDR3s were shared by the three libraries, and the proportion of CDR3s in HH + IHB_*shared*_ was higher than that of HH + CHB_*shared*_ (1.86% vs. 0.66%, *p* < 2.2E-16, *OR* = 4.325, 95%CI: 4.116–4.545; Figure 2D). In addition, the shared proportion of V(D)J rearranged patterns and CDR3 showed no significant difference between HH + IHB_*shared*_ and HH + CHB_*shared*_ in the IgG repertoires (V(D)J rearrangements: 12.05% vs.12.21%, *p* = 0.67, *OR* = 1.014, 95%CI: 0.952–1.079; CDR3s: 2.21% vs.1.92%, *p* < 2.2E-16, *OR* = 1.152, 95%CI: 1.090–1.219; Figures 2E,F). Taken together, there are more clones shared between the IgM repertories of HH and IHB library, and the extra shared clones could be due to the more same unique CDR3s shared between them.

### The V(D)J Gene Usage

**FIGURE 3:**
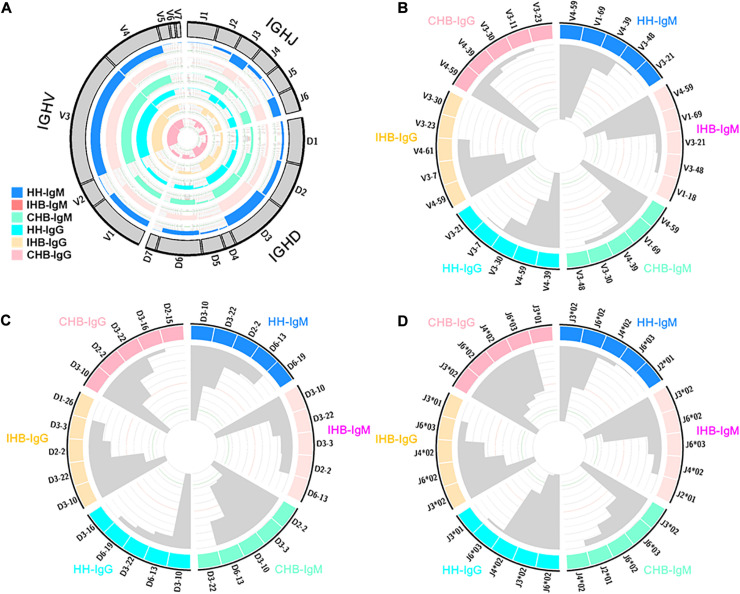
**(A)** The usage of VDJ gene families in IgM and IgG repertoires of the HH, IHB and CHB library. The outsider arcs represent the VDJ gene families, and the histograms inside the circle represent the usage of each gene in the IgM repertoires of HH (DogerBlue), IHB (MistyRose), and CHB (Aquamarine) and in IgG repertoires of HH (cyan), IHB (Moccasin), and CHB (pink). **(B)** The top 5 preferred used IGHV gene subgroups in the IgM and IgG repertoires of the three libraries. The histograms inside the circle represent the usage of each gene subgroups. **(C)** The top 5 preferred used IGHD gene subgroups in the IgM and IgG repertoires of the three libraries. The histograms inside the circle represent the usage of each gene subgroups. **(D)** The top 5 preferred used IGHJ gene alleles in the IgM and IgG repertoires of the three libraries. The histograms inside the circle represent the usage of each gene alleles.

To find the preferentially utilized V(D)J genes in the repertoires, the usage of V(D)J genes were calculated (Supplementary Tables 1–6). In heavy chains repertoires, 7 IGHV gene families containing 52 IGHV genes were observed, of which IGHV1, IGHV3 and IGHV4 family were frequently used, together accounting for more than 90% in all libraries (Figure 3A). Moreover, IGHV4-59 and IGHV1-69 were the top two frequently used genes in IgM repertoires of all three libraries, together accounting for about 20% of the total (Figure 3B). In IgG repertoires, IGHV4-39 and IGHV4-59 were frequently used in HH, with a rate of 10.18 and 8.27%, respectively (Figure 3B). However, in IHB and CHB, IGHV4-59 was the most frequently used gene with a rate of 12.86 and 12.74%, respectively, of which the usage was much higher than IGHV4-39 (IHB: 4.27%, CHB: 5.79%). Besides, IGHV3-7 was also frequently used in IHB with a rate nearly 10%, but which was less used in HH and CHB (IHB vs. CHB vs. HH: 9.86% vs. 4.15% vs. 5.44%).

There are 21 IGHD genes belong to 7 gene families in the heavy chain repertoires, among which IGHD2, IGHD3 and IGHD6 family were frequently used (Figure 3A). In IgM repertoires, the preferred used IGHD genes were IGHD3-10 and IGHD3-22 in both HH and IHB, together accounting for more than 20% in these two libraries. However, the preferred used IGHD genes were IGHD2-2 and IGHD3-3 in CHB, with a rate of 11.47 and 10.69%, respectively (Figure 3C). In IgG repertoires, IGHD3-10, IGHD6-13, and IGHD3-22 were the top three frequently used genes in HH, and the top three were IGHD3-10, IGHD2-2, and IGHD3-22 in IHB and CHB. IGHD3-10 was the most frequently used gene in all three libraries (Figure 3C).

When calculating the usage of IGHJ genes, the information of gene alleles was included. There are 13 IGHJ alleles belong to 6 gene families. The most frequently used IGHJ family were IGHJ3 and IGHJ6 in all libraries (Figure 3A). The frequently used IGHJ alleles were IGHJ3^∗^02, IGHJ6^∗^02, IGHJ4^∗^02, and IGHJ6^∗^03 in the IgM repertoires of HH and IHB library, and IGHJ3^∗^02, IGHJ6^∗^03, IGHJ6^∗^02, and IGHJ2^∗^01 in CHB. IGHJ3^∗^02 was extremely biased used in all the three libraries, accounting for more than 30% of the total. Interestingly, IGHJ6^∗^02 and IGHJ6^∗^03 were almost equally frequently used in CHB (IGHJ6^∗^02 vs. IGHJ6^∗^03: 14.52% vs. 18.53%); but in HH and IHB library, the frequency of IGHJ6^∗^02 was about twice higher than that of IGHJ6^∗^03 (HH: 22.68% vs. 10.20%; IHB: 25.32% vs. 12.51%). Incidentally, these two alleles have two different amino acids in sequences according to the IMGT database. In IgG repertoires, the biased used IGHJ alleles were very similar among the three libraries that were IGHJ6^∗^02, IGHJ3^∗^02, IGHJ4^∗^02, and IGHJ6^∗^03 (Figure 3D).

Taken together, the preferred used IGHV genes were similar among the three libraries in IgM repertoires, but the biased used IGHD genes and IGHJ alleles were different in CHB compared with HH and IHB. Relatively, the biased used VDJ genes were similar between the IgG repertoires of IHB and CHB library.

### Somatic Hypermutations in V Segments

The somatic hypermutations in V gene segments were analyzed by comparing the sequencing data with the germline genes from the IMGT database. A threshold value of ≥ 90% V gene identity was selected. In IgM repertoires, more than 90% sequences shared ≥ 90% identity with the germline V genes that was 94.2% in HH, 94.2% in IHB, and 93.1% in CHB. However, the proportion of sequences carrying germline-like V genes decreased significantly in the IgG repertoires, with a rate not more than 60% (59.7% in HH, 56.9% in IHB, and 57.5% in CHB). Then, the frequency of amino acid mutations in CDR1 and CDR2 regions were further calculated. In IgM repertoires, the proportion of sequences with amino acid changes in CDR1 and CDR2 regions demonstrated no significant difference among the three libraries (CDR1: 22.71% in HH, ac22.17% in IHB, and 25.95% in CHB; CDR2: 41.34% in HH, 41.65%in IHB, and 45.39% in CHB). In IgG repertoires, the proportion of sequences with mutations in CDR1 region also showed no significant difference among the three libraries (CDR1: 79.58% in HH, 83.37% in IHB, and 79.53% in CHB). Besides, the proportion of sequences with mutations in CDR2 regions was 82.91% in HH, 85.58% in IHB and 87.50% in CHB, which showed relatively higher difference only between CHB and HH (*p* < 2.2E-16, *OR* = 1.442, 95%CI: 1.413–1.471, Supplementary Table 7). Altogether, the somatic mutation level in the repertoires of HBV carriers did not show significant difference from that of the healthy adults, indicating the HBV infection might not induce significantly higher frequency of somatic mutations in antibody sequences.

### The Characteristics of CDR3 Regions

**FIGURE 4:**
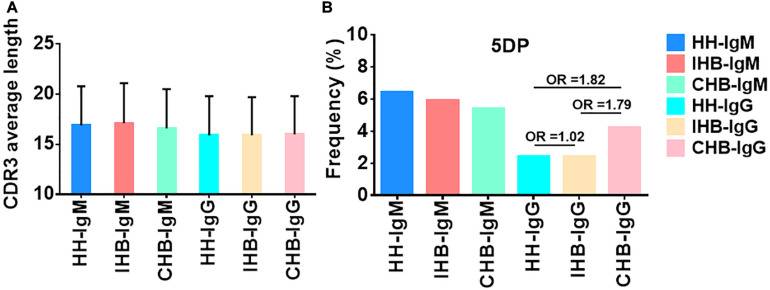
**(A)** The average length of CDR3s in IgM and IgG repertoires of the HH, IHB and CHB library. **(B)** The occurrence of P nucleotides additions at 5′-end of D genes (5DP) in the IgM and IgG repertoires of HH, IHB, and CHB library.

The characteristics of the CDR3 regions were analyzed, including the length of distribution, the amino acid usage of CDR3s and the occurrence and average length of junctional modifications. In our datasets, the length distribution of CDR3 ranged from 3 to 41 amino acids in heavy chains repertoires (Supplementary Figures 1A,B). For IgM repertoires, the CDR3 mean length was rounded to 17 amino acids for all three libraries, whereas it was 16 amino acids for the IgG repertoires of all libraries, approximately 1 amino acid shorter than that of the IgM repertoires (Figure 4A and Table 3). Moreover, the usage of amino acids in the CDR3 regions were analyzed. In the heavy chain repertoires of the three libraries, tyrosine, alanine, glycine, and aspartic acid were frequently used and the usage demonstrated no significant difference between libraries (Supplementary Figures 1C,D).

**TABLE 3:** The CDR3 mean length in all repertoires.

Repertoires		IgM	IgG
HH		16.9 ± 3.9	15.9 ± 3.9
IHB		17.1 ± 4.0	15.9 ± 3.8
CHB		16.6 ± 3.9	16.0 ± 3.8
Cohen’s *d*^*a*^	HH vs. IHB	0.03	0.004
	HH vs. CHB	0.09	0.03
	IHB vs. CHB	0.12	0.03

*^a^Student’s t-test and Cohen’s d value was used to measure the standardized difference between two means.*

The junctional modifications are the main mechanisms contributing to the CDR3 diversity, including additions of the palindromic nucleotides (P) and the non-template randomized nucleotides (N), as well as the deletion of nucleotides caused by exonuclease trimming (T). The characteristics of these modifications, including the occurrences and the average length, were analyzed, and showed no significant difference (Supplementary Figures 1E,F and Supplementary Tables 8–11) except for the occurrence of P addition at the 5’-end of D genes (5DP) in the IgG repertoires of CHB, which was almost twice that of the other two libraries (4.23% in CHB, 2.37% in HH and 2.41% in IHB, *OR*≈1.80, Figure 4B).

## Discussion

In this study, the high-throughput sequencing method was adapted to analyze the IgM and IgG repertoires of chronic HBV-infected individuals and healthy adults. After the analyses, we found that most of the characteristics were similar in the IgM and IgG repertoires between healthy adults and HBV carriers. Nevertheless, the diversity of unique clones decreased and some clones expanded in the IgM repertoire of chronic HBV carriers when compared with that of healthy adults and the inactive HBV carriers. Besides, there were more clones shared between the IgM repertoires of healthy adults and inactive HBV carriers than chronic carriers. The preferred used IGHD and IGHJ genes were similar between the IgM repertoire of healthy adults and inactive HBV carriers, but quite different from those in the chronic HBV carriers.

Variable definitions of unique antibody clones have been provided by different studies, such as unique nucleotide or amino acid sequences, or unique CDRs (Boyd et al., 2009; Glanville et al., 2009; Arnaout et al., 2011; Prabakaran et al., 2012; Chang et al., 2016; D’Angelo et al., 2018; Hong et al., 2018; Soto et al., 2019). The unique clone was defined as the sequence with unique V(D)J gene rearrangement or the sequence with unique CDR3 sequence that was used to identify the unique clone from the sequencing data. We found the diversity of unique clones in the IgM repertoire of chronic HBV carriers were smaller compared with the other two libraries. The extent of the reduction in diversity increased when the sequence number increased to 100,000 but decreased as the sequence number increased continuously, suggesting the calculation of the clone diversity depended on the sample size closely in the sampling survey of human antibody repertoire. Since the number of included individuals, the initial input cells, the sequencing depth, and the process of data analysis were same in all groups, the difference in diversity was less likely to result from the sampling or analytical errors. Generally, there are three primary mechanisms contributing to the repertoire diversity that are the V(D)J gene rearrangements, the junctional modifications in CDR3 regions and the somatic hypermutation (Furukawa et al., 1999; Glanville et al., 2009). However, no significant difference was found in the mutation level and the junctional modifications among the three libraries, suggesting that the diversity reduction in the IgM repertoire of chronic HBV carriers could result from the clone expansion. Polyclonal expansion would increase the possibility of capturing sequences belonging to the same cluster of unique clones and reduce the types of the unique clones. Although the sampling size in our study was insufficient to detect all the expand clones, the expansion of some clusters of unique clones still could be observed in our study. On the other hand, the diversity of unique clones in IgG repertoires demonstrated no significant difference among the three libraries. Although the occurrence of 5DP in the repertoire of chronic HBV carriers was more than the other two libraries, the contribution of this modification to increase the clone diversity was small since the occurrence of 5DP itself was lower than other types of junctional modifications in CDR3 regions. Altogether, these results might indicate that the high level of HBV replication was likely to induce the expansion of some IgM clones that reduced the diversity of the unique clones in IgM repertoires.

It has been suggested that the presence of the shared clones among individuals did not occur by chance alone, although it accounted for a small part of antibody repertoires (Galson et al., 2016; Miho et al., 2019; Soto et al., 2019). It has been suggested the converging CDR3s might be derived from the convergent V(D)J gene recombination or the convergent selection by the same antigen (Venturi et al., 2008; Venturi et al., 2011; Pogorelyy et al., 2018; Fink, 2019). In our study, more shared CDR3s were found between the IgM repertoires of healthy adults and inactive HBV carriers. Meanwhile, the preferred used IGHV, IGHD, and IGHJ genes were found to be similar between the two libraries, indicating that the extra shared CDR3s might result from the similar selection of VDJ genes that were more prone to result in the same amino acid sequences. Conversely, the different biased usage of IGHD and IGHJ genes contributed to the deviation of clones in the IgM repertoire of chronic HBV carriers.

Somatic hyermutation is a critical mechanism to diversify the antibody clones and fulfills a central role in antibody affinity maturation (Di Noia and Neuberger, 2007; Hwang et al., 2015; Kitaura et al., 2017). In general, antibodies undergo iterative mutations and stepwise antigen-mediated selections to develop the antibodies with high antigen affinity (Di Noia and Neuberger, 2007; Victora and Nussenzweig, 2012; Hoehn et al., 2016). Thus, high level of mutations were commonly seen in the antibody repertoires after virus infection or vaccinations (Laserson et al., 2014; Tan et al., 2014; Wang et al., 2014; Cortina-Ceballos et al., 2015; Wendel et al., 2017), including the BCR repertoires after hepatitis B vaccinations (Galson et al., 2015; Galson et al., 2016). However, little change in the somatic mutation level was observed in antibody repertoires with some chronic virus infections, such as HIV-1, Epstein-Barr virus (Yin et al., 2013; Wang et al., 2014). Additionally, the frequency of mutations in the V segments also showed no significant difference between the three libraries in our study, suggesting that the HBV infection might not induce intensive antibody mutations in the phase of chronic infection.

In-depth analyses of the antibody repertoires in patients with infectious diseases could provide a better understanding of the interaction between human immune system and infectious pathogens. Our study showed that low serum HBV level might not induce significant changes in BCR repertoires, as the characteristics of IgM and IgG repertoires were similar to that of healthy adults. Importantly, significant changes were observed in IgM repertoires under the impact of high titer of HBV, indicating that the B cell repertoires could respond to the high titer of HBV although insufficient to eliminate the virus during the chronic infection phase.

## Data Availability Statement

The sequencing data is available in NCBI SRA database (https://www.ncbi.nlm.nih.gov/sra). The access number is PRJNA578020 (https://www.ncbi.nlm.nih.gov/bioproject/PRJNA 578020).

## Ethics Statement

The studies involving human participants were reviewed and approved by the Second Affiliated Hospital of Fujian Medical University. The patients/participants provided their written informed consent to participate in this study.

## Author Contributions

BH and TY conceived and designed the project. CH, QLi, QLiu, and XH carried out the experiments. BH, LS, AL, LixW, and CW analyzed the data. BH, LizW, and TY wrote the manuscript with input from all co-authors. All authors contributed to the article and approved the submitted version.

## Conflict of Interest

The authors declare that the research was conducted in the absence of any commercial or financial relationships that could be construed as a potential conflict of interest.

## Footnotes

1 www.imgt.org

2 https://www.ncbi.nlm.nih.gov/sra/?term=PRJNA578020
