# Supplementary material for: Decrease of Clone Diversity in IgM Repertoires of HBV Chronically Infected Individuals With High Level of Viral Replication
Source: Front Microbiol. 2021 Jan 15;11:615669. doi: 10.3389/fmicb.2020.615669 (PMC7843509; doi:10.3389/fmicb.2020.615669)
Supplement: Supplementary file 1 [file Image_1.pdf]

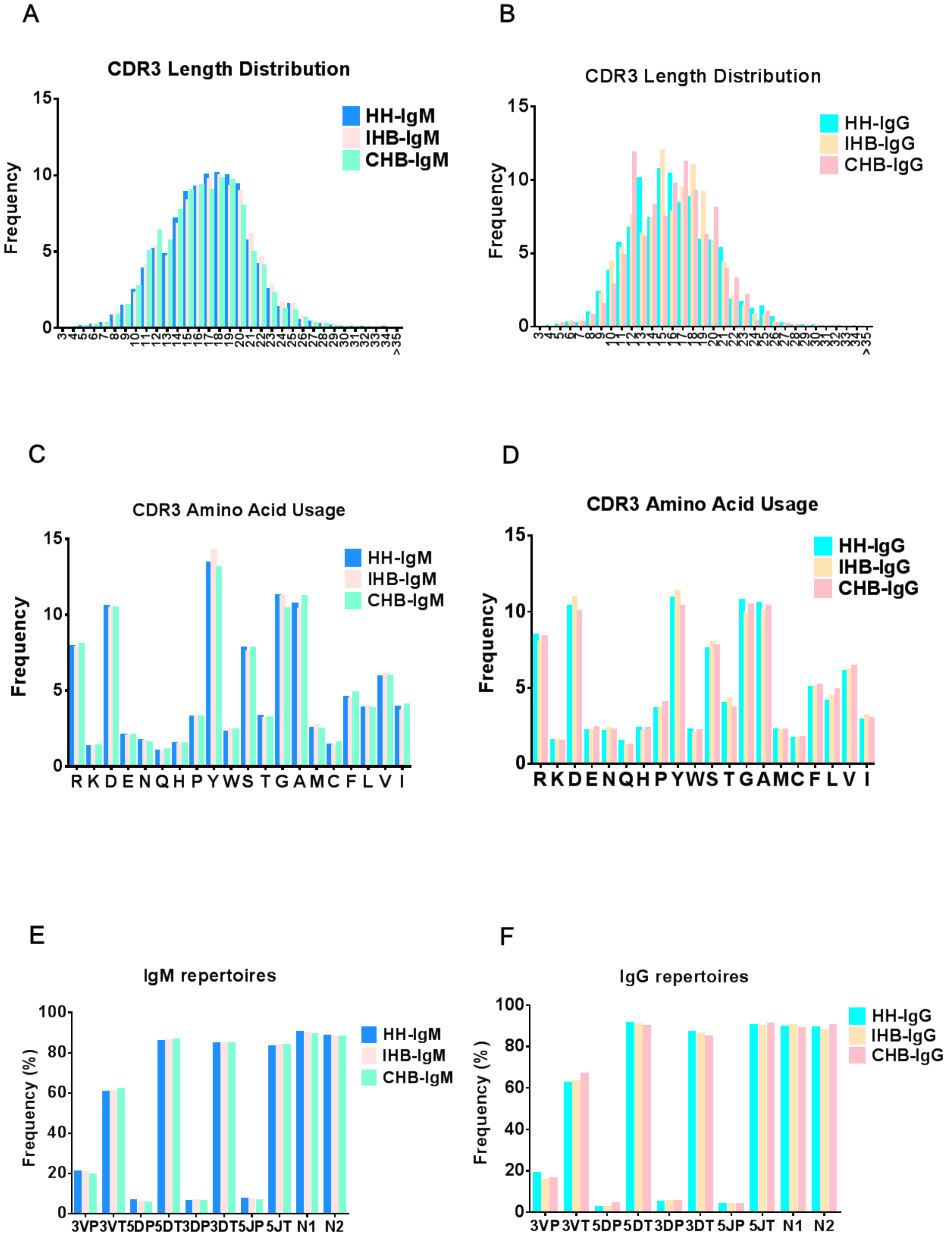

Supplementary Figure 1|(A) The length distribution of CDR3 sequences in IgM repertoires of the three libraries. **(B)** The length distribution of CDR3 sequences in IgG repertoires of the three libraries. **(C)** The amino acid usage of CDR3 sequences in IgM repertoires of the three libraries. **(D)** The amino acid usage of CDR3 sequences in IgG repertoires of the three libraries. **(E)** The occurrence of different types of junctional modifications in IgM repertoires of the three libraries. 3VP and 3VT: the palindromic nucleotides (P) additions and exonuclease trimmings observed at 3'-end of V regions, respectively; 5DP and 5DT: the P additions and exonuclease trimmings observed at 5'-end of D genes, respectively; 3DP and 3DT: the P additions and exonuclease trimmings

observed at 3'-end of D genes, respectively; 5JP and 5JT: the P additions and exonuclease trimmings observed at 5'-end of J genes, respectively; N1: the non-template randomized nucleotides (N) additions happened at the region between the 3'-end of V gene and the 5'-end of D gene; N2: N additions happened at the region between the3'-end of D gene and the 5'-end of J gene. **(F)** The occurrence of different types of junctional modifications in IgG repertoires of the three libraries.
