# Supplementary material for: Decrease of Clone Diversity in IgM Repertoires of HBV Chronically Infected Individuals With High Level of Viral Replication
Source: Front Microbiol. 2021 Jan 15;11:615669. doi: 10.3389/fmicb.2020.615669 (PMC7843509; doi:10.3389/fmicb.2020.615669)
Supplement: Supplementary file 2 [file Table_1.pdf]

Supplementary Table 1. The Usage of IGHV Genes in IgM Repertoires

HH-IgM	Frequency (%)	IHB-IgM	Frequency (%)	CHB-IgM	Frequency (%)
IGHV4-59	10.62	IGHV4-59	11.49	IGHV4-59	11.52
IGHV1-69	10.36	IGHV1-69	9.65	IGHV1-69	7.95
IGHV4-39	5.97	IGHV3-21	5.45	IGHV4-39	5.58
IGHV3-48	4.73	IGHV3-48	5.3	IGHV3-30	5.48
IGHV3-21	4.61	IGHV1-18	4.99	IGHV3-48	5.16
IGHV3-30	4.6	IGHV3-30	4.95	IGHV3-21	4.94
IGHV3-23	4.52	IGHV4-39	4.68	IGHV1-18	4.44
IGHV1-18	4.43	IGHV3-23	4.52	IGHV3-23	4.38
IGHV4-34	4.31	IGHV4-34	4.5	IGHV4-61	4.15
IGHV1-46	3.46	IGHV4-61	3.64	IGHV4-34	3.83
IGHV4-61	3.25	IGHV1-46	3.55	IGHV1-46	3.31
IGHV3-7	2.91	IGHV3-7	3.23	IGHV4-4	3.26
IGHV3-33	2.72	IGHV3-33	3.01	IGHV3-7	3.09
IGHV3-11	2.62	IGHV3-11	2.69	IGHV3-11	2.8
IGHV4-4	2.6	IGHV4-4	2.4	IGHV4-31	2.41
IGHV4-31	2.54	IGHV3-9	2.33	IGHV3-33	2.39
IGHV3-9	2.33	IGHV1-8	2.17	IGHV3-9	1.85
IGHV1-8	2.08	IGHV4-31	1.82	IGHV7-4-1	1.75
IGHV3-53	1.92	IGHV7-4-1	1.82	IGHV3-53	1.72
IGHV3-74	1.69	IGHV3-53	1.72	IGHV3-74	1.69
IGHV3-49	1.62	IGHV1-3	1.61	IGHV3-66	1.62
IGHV7-4-1	1.51	IGHV3-49	1.52	IGHV3-30-3	1.61
IGHV1-3	1.39	IGHV2-5	1.26	IGHV1-8	1.54
IGHV4-38-2	1.25	IGHV3-74	1.25	IGHV1-3	1.5
IGHV2-5	1.24	IGHV3-66	1.14	IGHV3-49	1.23
IGHV1-2	1.15	IGHV1-2	1.09	IGHV1-2	1.11
IGHV3-30-3	1.09	IGHV3-30-3	1.01	IGHV3-13	1.07
IGHV3-66	1.08	IGHV3-13	0.72	IGHV5-10-1	0.84
IGHV1-24	0.9	IGHV2-70	0.69	IGHV4-38-2	0.79
IGHV5-10-1	0.84	IGHV1-24	0.56	IGHV3-43	0.71
IGHV3-13	0.64	IGHV3-15	0.56	IGHV1-24	0.69
IGHV2-26	0.5	IGHV3-43	0.53	IGHV2-70	0.62
IGHV2-70	0.47	IGHV3-20	0.5	IGHV3-20	0.58
IGHV3-64	0.45	IGHV5-10-1	0.48	IGHV4-30-4	0.54
IGHV3-20	0.44	IGHV3-72	0.43	IGHV2-5	0.51
IGHV4-30-2	0.44	IGHV4-30-4	0.42	IGHV3-15	0.48
IGHV3-15	0.42	IGHV2-26	0.38	IGHV3-64	0.48
IGHV3-43	0.38	IGHV5-51	0.34	IGHV6-1	0.47
IGHV4-30-4	0.38	IGHV3-64	0.29	IGHV3-72	0.38
IGHV5-51	0.34	IGHV6-1	0.26	IGHV2-26	0.28
IGHV3-72	0.31	IGHV3-73	0.24	IGHV4-30-2	0.28
IGHV1-58	0.19	IGHV4-30-2	0.23	IGHV3-73	0.23
IGHV6-1	0.19	IGHV1-58	0.22	IGHV5-51	0.2
IGHV3-73	0.17	IGHV4-38-2	0.17	IGHV1-58	0.18
IGHV3-43D	0.16	IGHV3-NL1	0.1	IGHV3-NL1	0.12
IGHV3-NL1	0.08	IGHV3-64D	0.06	IGHV3-43D	0.11
IGHV3-64D	0.04	IGHV4-28	0.03	IGHV3-64D	0.09
IGHV1-69-2	0.03	IGHV1-45	0.03	IGHV1-45	0.02
IGHV4-28	0.01	IGHV2-70D	0.02	IGHV4-28	0.02
IGHV1-45	0.01	IGHV3-43D	0.01	IGHV1-69-2	0.01
IGHV2-70D	0.00[a]	IGHV1-69-2	0.00	IGHV2-70D	0.00

a: The gene frequency was less than 0.001.
