# Supplementary material for: Decrease of Clone Diversity in IgM Repertoires of HBV Chronically Infected Individuals With High Level of Viral Replication
Source: Front Microbiol. 2021 Jan 15;11:615669. doi: 10.3389/fmicb.2020.615669 (PMC7843509; doi:10.3389/fmicb.2020.615669)
Supplement: Supplementary file 3 [file Table_2.pdf]

Supplementary Table 2. The Usage of IGHV Genes in IgG Repertoires

HH-IgG	Frequency (%)	IHB-IgG	Frequency (%)	CHB-IgG	Frequency (%)
IGHV4-39	10.18	IGHV4-59	12.86	IGHV4-59	12.74
IGHV4-59	8.27	IGHV3-7	9.86	IGHV4-39	5.79
IGHV3-30	6.37	IGHV4-61	6.31	IGHV3-30	5.31
IGHV3-7	5.44	IGHV3-23	5.08	IGHV3-11	5.19
IGHV3-21	5.37	IGHV3-30	5.07	IGHV3-23	4.88
IGHV3-48	5.34	IGHV3-48	4.90	IGHV4-61	4.46
IGHV3-23	4.55	IGHV3-21	4.72	IGHV1-69	4.40
IGHV1-69	4.43	IGHV4-39	4.27	IGHV3-7	4.15
IGHV1-18	4.25	IGHV1-69	3.75	IGHV3-21	3.80
IGHV3-74	3.85	IGHV4-34	3.23	IGHV4-31	3.67
IGHV1-46	3.50	IGHV1-46	3.20	IGHV3-48	3.56
IGHV2-5	3.14	IGHV3-9	3.04	IGHV1-18	2.79
IGHV3-33	2.81	IGHV3-74	2.97	IGHV4-4	2.70
IGHV3-9	2.74	IGHV4-4	2.91	IGHV4-34	2.69
IGHV3-11	2.73	IGHV3-11	2.58	IGHV2-5	2.54
IGHV4-61	2.49	IGHV2-5	2.13	IGHV3-66	2.40
IGHV4-34	1.86	IGHV3-33	1.75	IGHV4-30-4	2.30
IGHV3-49	1.69	IGHV1-18	1.69	IGHV3-53	2.20
IGHV1-8	1.67	IGHV3-49	1.61	IGHV3-49	2.16
IGHV4-4	1.50	IGHV4-31	1.49	IGHV3-33	2.07
IGHV4-38-2	1.41	IGHV3-15	1.42	IGHV3-74	1.82
IGHV3-15	1.38	IGHV3-72	1.38	IGHV5-10-1	1.62
IGHV7-4-1	1.26	IGHV3-53	1.31	IGHV1-46	1.58
IGHV3-64	1.17	IGHV2-70	1.23	IGHV7-4-1	1.50
IGHV3-43	1.14	IGHV3-66	1.13	IGHV3-15	1.41
IGHV3-72	1.12	IGHV5-10-1	1.00	IGHV1-8	1.34
IGHV3-53	1.08	IGHV3-13	0.79	IGHV3-9	1.32
IGHV4-31	1.04	IGHV3-64	0.75	IGHV1-3	0.94
IGHV3-66	0.96	IGHV5-51	0.73	IGHV3-30-3	0.93
IGHV3-30-3	0.87	IGHV7-4-1	0.71	IGHV4-38-2	0.92
IGHV5-51	0.85	IGHV3-30-3	0.68	IGHV4-30-2	0.91
IGHV1-2	0.76	IGHV3-43	0.66	IGHV3-13	0.86
IGHV5-10-1	0.62	IGHV1-2	0.65	IGHV1-2	0.66
IGHV1-3	0.60	IGHV1-8	0.61	IGHV3-64	0.62
IGHV2-70	0.50	IGHV1-24	0.60	IGHV3-43	0.61
IGHV1-24	0.45	IGHV3-20	0.55	IGHV3-73	0.55
IGHV3-13	0.42	IGHV1-3	0.47	IGHV3-72	0.46
IGHV4-30-4	0.40	IGHV2-26	0.43	IGHV5-51	0.43
IGHV6-1	0.38	IGHV3-73	0.39	IGHV6-1	0.43
IGHV3-20	0.34	IGHV4-30-4	0.37	IGHV1-24	0.33
IGHV3-73	0.30	IGHV4-38-2	0.20	IGHV2-26	0.30
IGHV2-26	0.27	IGHV6-1	0.19	IGHV2-70	0.21
IGHV3-43D	0.15	IGHV4-30-2	0.11	IGHV3-20	0.10
IGHV4-30-2	0.10	IGHV1-58	0.08	IGHV3-64D	0.09
IGHV1-58	0.10	IGHV3-64D	0.07	IGHV3-NL1	0.08
IGHV3-64D	0.06	IGHV3-NL1	0.05	IGHV1-58	0.06
IGHV3-NL1	0.05	IGHV3-43D	0.03	IGHV3-43D	0.06
IGHV4-28	0.02	IGHV4-28	0.02	IGHV4-28	0.02
IGHV1-69-2	0.01	IGHV1-45	0.01	IGHV1-45	0.01
IGHV1-45	0.01	IGHV1-69-2	0.00	IGHV1-69-2	0.01
IGHV2-70D	0.00	IGHV2-70D	0.00	IGHV2-70D	0.00
