# Supplementary material for: Decrease of Clone Diversity in IgM Repertoires of HBV Chronically Infected Individuals With High Level of Viral Replication
Source: Front Microbiol. 2021 Jan 15;11:615669. doi: 10.3389/fmicb.2020.615669 (PMC7843509; doi:10.3389/fmicb.2020.615669)
Supplement: Supplementary file 4 [file Table_3.pdf]

Supplementary Table 3. The Usage of IGHD Genes in IgM Repertoires

HH-IgM	Frequency (%)	IHB-IgM	Frequency (%)	CHB-IgM	Frequency (%)
IGHD3-10	15.62	IGHD3-10	13.57	IGHD2-2	11.47
IGHD3-22	11.30	IGHD3-22	9.58	IGHD3-3	10.69
IGHD2-2	8.39	IGHD3-3	9.03	IGHD3-10	10.62
IGHD6-13	8.12	IGHD2-2	8.95	IGHD6-13	8.96
IGHD6-19	6.56	IGHD6-13	8.20	IGHD3-22	7.73
IGHD2-15	5.99	IGHD6-19	6.02	IGHD6-19	6.09
IGHD3-3	5.98	IGHD2-15	5.83	IGHD1-26	5.60
IGHD1-26	5.65	IGHD1-26	5.71	IGHD2-15	5.20
IGHD3-9	5.16	IGHD3-16	4.81	IGHD3-16	4.58
IGHD3-16	4.53	IGHD3-9	4.56	IGHD6-6	4.46
IGHD4-17	4.11	IGHD4-17	3.66	IGHD2-21	3.45
IGHD5-18	3.64	IGHD6-6	3.47	IGHD4-17	3.44
IGHD5-12	3.46	IGHD5-18	3.31	IGHD5-12	3.36
IGHD2-21	3.11	IGHD2-21	3.11	IGHD3-9	3.18
IGHD6-6	2.27	IGHD5-12	3.08	IGHD5-18	2.90
IGHD2-8	1.82	IGHD2-8	2.07	IGHD2-8	2.61
IGHD1-1	1.61	IGHD1-1	1.92	IGHD1-1	2.09
IGHD1-7	0.91	IGHD1-7	1.04	IGHD1-7	1.21
IGHD6-25	0.73	IGHD6-25	0.86	IGHD7-27	0.97
IGHD7-27	0.62	IGHD7-27	0.72	IGHD6-25	0.76
IGHD1-20	0.40	IGHD1-20	0.50	IGHD1-20	0.64
