# Supplementary material for: Decrease of Clone Diversity in IgM Repertoires of HBV Chronically Infected Individuals With High Level of Viral Replication
Source: Front Microbiol. 2021 Jan 15;11:615669. doi: 10.3389/fmicb.2020.615669 (PMC7843509; doi:10.3389/fmicb.2020.615669)
Supplement: Supplementary file 5 [file Table_4.pdf]

Supplementary Table 4. The Usage of IGHD Genes in IgG Repertoires

HH-IgG	Frequency (%)	IHB-IgG	Frequency (%)	CHB-IgG	Frequency (%)
IGHD3-10	13.28	IGHD3-10	13.77	IGHD3-10	14.51
IGHD3-22	8.01	IGHD3-22	11.70	IGHD2-2	10.09
IGHD2-2	7.05	IGHD2-2	8.34	IGHD3-22	7.90
IGHD6-13	8.28	IGHD3-3	7.50	IGHD3-16	6.13
IGHD6-19	7.78	IGHD1-26	6.11	IGHD2-15	5.76
IGHD2-15	6.77	IGHD2-21	5.53	IGHD3-3	5.71
IGHD3-3	6.01	IGHD3-16	5.35	IGHD6-13	5.61
IGHD1-26	6.06	IGHD6-13	5.29	IGHD6-6	5.50
IGHD3-9	3.54	IGHD6-19	5.24	IGHD6-19	5.18
IGHD3-16	7.47	IGHD2-15	5.21	IGHD1-26	4.91
IGHD4-17	3.38	IGHD3-9	4.52	IGHD2-21	4.80
IGHD5-18	2.73	IGHD5-12	3.42	IGHD3-9	3.94
IGHD5-12	4.11	IGHD2-8	3.23	IGHD5-12	3.61
IGHD2-21	4.60	IGHD4-17	3.09	IGHD2-8	3.49
IGHD6-6	1.88	IGHD1-1	3.01	IGHD5-18	3.41
IGHD2-8	4.10	IGHD6-6	2.64	IGHD4-17	3.20
IGHD1-1	2.01	IGHD5-18	2.38	IGHD1-1	2.33
IGHD1-7	0.51	IGHD1-7	1.31	IGHD6-25	1.46
IGHD6-25	1.11	IGHD1-20	0.87	IGHD7-27	1.27
IGHD7-27	0.78	IGHD7-27	0.83	IGHD1-7	0.65
IGHD1-20	0.56	IGHD6-25	0.66	IGHD1-20	0.52
