# Supplementary material for: Decrease of Clone Diversity in IgM Repertoires of HBV Chronically Infected Individuals With High Level of Viral Replication
Source: Front Microbiol. 2021 Jan 15;11:615669. doi: 10.3389/fmicb.2020.615669 (PMC7843509; doi:10.3389/fmicb.2020.615669)
Supplement: Supplementary file 6 [file Table_5.pdf]

Supplementary Table 5. The Usage of IGHJ Genes in IgM Repertoires

**HH-IgM**	**Frequency** (%)	**IHB-IgM**	**Frequency** (%)	**CHB-IgM**	**Frequency** (%)
IGHJ3_02	33.98	IGHJ3_02	30.35	IGHJ3_02	37.96
IGHJ6_02	22.68	IGHJ6_02	25.32	IGHJ6_03	18.53
IGHJ4_02	11.15	IGHJ6_03	12.51	IGHJ6_02	14.52
IGHJ6_03	10.20	IGHJ4_02	10.66	IGHJ2_01	10.55
IGHJ2_01	9.97	IGHJ2_01	8.65	IGHJ4_02	8.10
IGHJ5_02	4.38	IGHJ5_02	4.85	IGHJ5_02	3.00
IGHJ3_01	2.71	IGHJ3_01	2.63	IGHJ3_01	2.80
IGHJ4_01	1.30	IGHJ4_01	1.26	IGHJ4_01	1.44
IGHJ1_01	1.21	IGHJ1_01	1.08	IGHJ1_01	0.93
IGHJ5_01	0.65	IGHJ6_04	0.79	IGHJ6_04	0.73
IGHJ4_03	0.64	IGHJ4_03	0.71	IGHJ4_03	0.64
IGHJ6_04	0.64	IGHJ5_01	0.64	IGHJ5_01	0.53
IGHJ6_01	0.49	IGHJ6_01	0.53	IGHJ6_01	0.27
