# Supplementary material for: Decrease of Clone Diversity in IgM Repertoires of HBV Chronically Infected Individuals With High Level of Viral Replication
Source: Front Microbiol. 2021 Jan 15;11:615669. doi: 10.3389/fmicb.2020.615669 (PMC7843509; doi:10.3389/fmicb.2020.615669)
Supplement: Supplementary file 7 [file Table_6.pdf]

Supplementary Table 6. The Usage of IGHJ Genes in IgG Repertoires

HH-IgG	Frequency (%)	IHB-IgG	Frequency (%)	CHB-IgG	Frequency (%)
IGHJ6_02	21.36	IGHJ3_02	22.60	IGHJ3_02	21.09
IGHJ3_02	21.11	IGHJ6_02	18.10	IGHJ6_02	17.32
IGHJ4_02	17.77	IGHJ4_02	15.16	IGHJ4_02	16.22
IGHJ6_03	10.12	IGHJ6_03	12.16	IGHJ6_03	15.42
IGHJ3_01	9.80	IGHJ3_01	11.06	IGHJ3_01	7.92
IGHJ2_01	6.51	IGHJ2_01	6.06	IGHJ2_01	6.79
IGHJ5_02	5.05	IGHJ5_02	5.81	IGHJ5_02	6.31
IGHJ4_01	3.36	IGHJ4_01	2.91	IGHJ4_01	3.27
IGHJ5_01	1.54	IGHJ5_01	2.21	IGHJ1_01	2.45
IGHJ1_01	1.21	IGHJ1_01	1.38	IGHJ5_01	1.22
IGHJ4_03	0.87	IGHJ6_04	1.11	IGHJ6_04	0.79
IGHJ6_01	0.67	IGHJ6_01	0.74	IGHJ4_03	0.64
IGHJ6_04	0.64	IGHJ4_03	0.71	IGHJ6_01	0.56
