# Supplementary material for: Decrease of Clone Diversity in IgM Repertoires of HBV Chronically Infected Individuals With High Level of Viral Replication
Source: Front Microbiol. 2021 Jan 15;11:615669. doi: 10.3389/fmicb.2020.615669 (PMC7843509; doi:10.3389/fmicb.2020.615669)
Supplement: Supplementary file 8 [file Table_7.pdf]

Supplementary Table 7. The Comparisons of the Somatic Hypermutation Levels in the Antibody Repertoires of the Three Libraries

	Library			*p, OR(95%CI)* [a]		
	**HH (%)**	**IHB (%)**	**CHB (%)**	**HH *vs.* IHB**	**HH *vs.* CHB**	**IHB *vs.* CHB**
**IgM repertoire**						
**V_{Iden} >90%**	94.21	94.18	93.08	0.471, 1.006(0.990,1.023)	<2.2E−16, 1.212(1.192,1.231)	<2.2E−16, 1.204(1.185,1.224)
**CDR1**	22.71	22.17	25.95	3.1E-11, 0.969(0.961,0.978)	<2.2E−16, 1.193(1.182,1.204)	<2.2E−16, 1.230(1.219,1.242)
**CDR2**	41.34	41.65	45.39	0.001, 1.013(1.005,1.021)	<2.2E−16, 1.179(1.170,1.189)	<2.2E−16, 1.164(1.155,1.173)
**IgG repertoire**						
**V_{Iden} >90%**	59.67	56.95	57.55	<2.2E−16, 1.118(1.102,1.135)	<2.2E−16, 1.091(1.076,1.107)	0.0004, 0.976(0.963,0.989)
**CDR1**	79.58	83.37	79.53	<2.2E−16, 1.287(1.263,1.310)	0.723, 0.997(0.980,1.014)	<2.2E−16, 0.775(0.761,0.788)
**CDR2**	82.91	85.58	87.50	<2.2E−16, 1.223 (1.199,1.247)	<2.2E−16, 1.442(1.413,1.471)	<2.2E−16, 1.179(1.156,1.202)

a: Calculated by the logistic regression.
