# Supplementary material for: Decrease of Clone Diversity in IgM Repertoires of HBV Chronically Infected Individuals With High Level of Viral Replication
Source: Front Microbiol. 2021 Jan 15;11:615669. doi: 10.3389/fmicb.2020.615669 (PMC7843509; doi:10.3389/fmicb.2020.615669)
Supplement: Supplementary file 9 [file Table_8.pdf]

Supplementary Table 8. The Occurrence of Junctional Modifications in IgM Repertoires

**Junctional Modification** [a]	**Occurrence (%)**									
	**3VP**	**5DP**	**3DP**	**5JP**	**N1**	**N2**	**3VT**	**5DT**	**3DT**	**5JT**
**HH**	20.92	6.40	6.21	7.26	90.17	88.21	60.62	85.65	84.44	83.16
**IHB**	20.67	5.88	6.40	6.80	89.71	87.95	61.02	86.11	84.54	83.72
**CHB**	19.40	5.44	6.18	6.38	89.10	87.80	61.85	86.32	84.70	83.84
p, [b]<br>***OR(95%CI)***<br>**(HH *vs.* IHB)**	0.001,<br>0.985<br>(0.976,0.994)	<2.2E−16,<br>0.914<br>(0.900,0.929)	6.0E-5,<br>1.033<br>(1.017,1.049)	<2.2E−16,<br>0.931<br>(0.917,0.945)	5.0E-15,<br>0.951<br>(0.939,0.963)	4.3E-5,<br>0.976<br>(0.964,0.987)	4.0E-5,<br>1.017<br>(1.009,1.024)	8.9E-12,<br>1.039<br>(1.028,1.050)	0.167,<br>1.007<br>(0.997,1.018)	1.0E-14,<br>1.041<br>(1.031,1.052)
p, [b]<br>***OR(95%CI)***<br>**(HH *vs.* CHB)**	<2.2E−16,<br>0.910<br>(0.901,0.919)	<2.2E−16,<br>0.841<br>(0.827,0.855)	0.645,<br>0.996<br>(0.980,1.013)	<2.2E−16,<br>0.869<br>(0.856,0.883)	<2.2E−16,<br>0.891<br>(0.879,0.903)	5.7E-10,<br>0.962<br>(0.950,0.974)	<2.2E−16,<br>1.053<br>(1.044,1.062)	<2.2E−16,<br>1.057<br>(1.045,1.069)	4.0E-4,<br>1.020<br>(1.009,1.031)	<2.2E−16,<br>1.050<br>(1.039,1.062)
p, [b]<br>***OR(95%CI)***<br>**(IHB *vs.* CHB)**	<2.2E−16,<br>0.924<br>(0.915,0.933)	<2.2E−16,<br>0.920<br>(0.905,0.936)	1.2E-5,<br>0.965<br>(0.949,0.980)	<2.2E−16,<br>0.934<br>(0.919,0.949)	<2.2E−16,<br>0.937<br>(0.925,0.949)	0.021,<br>0.986<br>(0.974,0.998)	<2.2E−16,<br>1.036<br>(1.027,1.044)	<2.2E−16,<br>1.017<br>(1.006,1.029)	0.026,<br>1.012<br>(1.001,1.023)	0.117,<br>1.009<br>(0.998,1.019)

a: Junctional modification: 3VP and 3VT: the palindromic nucleotides (P) additions and exonuclease trimmings observed at 3′-end of V regions, respectively; 5DP and 5DT: the P additions and exonuclease trimmings observed at 5′-end of D genes, respectively; 3DP and 3DT: the P additions and exonuclease trimmings observed at 3′-end of D genes, respectively; 5JP and 5JT: the P additions and exonuclease trimmings observed at 5′-end of J genes, respectively; N1: the non-template randomized nucleotides (N) additions happened at the region between the 3′-end of V gene and the 5′-end of D gene; N2: N additions happened at the region between the3′-end of D gene and the 5′-end of J gene.
b: Calculated by the logistic regression.
