# Supplementary material for: Decrease of Clone Diversity in IgM Repertoires of HBV Chronically Infected Individuals With High Level of Viral Replication
Source: Front Microbiol. 2021 Jan 15;11:615669. doi: 10.3389/fmicb.2020.615669 (PMC7843509; doi:10.3389/fmicb.2020.615669)
Supplement: Supplementary file 10 [file Table_9.pdf]

Supplementary Table 9. The Occurrence of Junctional Modifications in IgG Repertoires

Junctional Modification	Occurrence (%)									
	**3VP**	**5DP**	**3DP**	**5JP**	**N1**	**N2**	**3VT**	**5DT**	**3DT**	**5JT**
**HH**	18.72	2.37	4.97	3.73	89.35	89.15	62.38	91.19	87.04	90.28
**IHB**	15.65	2.41	5.12	3.61	90.09	87.75	62.95	90.43	86.37	89.73
**CHB**	16.10	4.23	5.27	3.91	88.75	90.20	66.68	89.80	84.62	91.10
***p*,[a] *OR(95%CI)* (HH *vs.* IHB)**	<2.2E−16, 0.805 (0.790,0.821)	0.425, 1.019 (0.973,1.068)	0.058, 1.032 (0.999,1.066)	0.075, 0.966 (0.930,1.003)	2.1E-11, 1.083 (1.058,1.109)	<2.2E−16, 0.872 (0.853,0.892)	0.001, 1.025 (1.010,1.040)	3.8E-13, 0.913 (0.890,0.935)	6.0E-08, 0.944 (0.924,0.964)	5.3E-07, 0.941 (0.919,0.964)
***p*,[a] *OR(95%CI)* (HH *vs.* CHB)**	<2.2E−16, 0.833 (0.818,0.849)	<2.2E−16, 1.822 (1.748,1.900)	0.0001, 1.064 (1.030,1.098)	0.009, 1.050 (1.012,1.089)	8.0E-08, 0.940 (0.919,0.962)	<2.2E−16, 1.121 (1.095,1.147)	<2.2E−16, 1.207 (1.189,1.225)	<2.2E−16, 0.850 (0.830,0.871)	<2.2E−16, 0.819 (0.803,0.836)	1.7E-15, 1.103 (1.077,1.130)
***p*,[a] *OR(95%CI)* (IHB *vs.* CHB)**	0.0003, 1.034 (1.016,1.054)	<2.2E−16, 1.788 (1.720,1.859)	0.05, 1.031 (1.000,1.062)	4.0E-06, 1.087 (1.049,1.126)	<2.2E−16, 0.868 (0.852,0.885)	<2.2E−16, 1.285 (1.258,1.313)	<2.2E−16, 1.178 (1.162,1.195)	7.0E-10, 0.932 (0.911,0.953)	<2.2E−16, 0.868 (0.852,0.885)	<2.2E−16, 1.172 (1.146,1.199)

a: Calculated by the logistic regression.
