# Supplementary material for: Decrease of Clone Diversity in IgM Repertoires of HBV Chronically Infected Individuals With High Level of Viral Replication
Source: Front Microbiol. 2021 Jan 15;11:615669. doi: 10.3389/fmicb.2020.615669 (PMC7843509; doi:10.3389/fmicb.2020.615669)
Supplement: Supplementary file 11 [file Table_10.pdf]

Supplementary Table 10. The Average Length of Nucleotides Added and Trimmed in the Junctional Regions of IgM Repertoires

Junctional Modification	Average Length (nt)									
	3VP	5DP	3DP	5JP	N1	N2	3VT	5DT	3DT	5JT
**HH**	1.57± 0.75	1.64± 0.83	1.36± 0.71	1.47±0.80	7.03± 4.83	6.49± 4.87	2.25± 1.59	6.62± 4.74	6.22±4.06	6.01± 4.77
**IHB**	1.55± 0.77	1.59± 0.75	1.39± 0.72	1.46± 0.80	6.95± 4.88	6.67± 5.05	2.25 ±1.60	6.66 ±4.85	6.23± 4.12	6.12± 4.78
**CHB**	1.56± 0.74	1.68 ±0.92	1.37± 0.72	1.45± 0.74	6.80± 4.81	6.34± 4.77	2.30 ±1.62	6.81± 4.97	6.19 ±4.14	5.95± 4.78
Cohen's d [a] **(HH *vs.* IHB)**	0.03	0.08	0.05	0.01	0.004	0.01	0.003	0.002	0.001	0.01
Cohen's d [a] **(HH *vs.* CHB)**	0.01	0.04	0.01	0.03	0.01	0.01	0.01	0.01	0.002	0.003
Cohen's d [a] **(IHB *vs.* CHB)**	0.02	0.12	0.04	0.02	0.01	0.01	0.01	0.01	0.003	0.01

a: Calculated by Student's t test; *Cohen's d* Value: when d ≥ 0.20 , the difference were considered to be significant.
