# Supplementary material for: Decrease of Clone Diversity in IgM Repertoires of HBV Chronically Infected Individuals With High Level of Viral Replication
Source: Front Microbiol. 2021 Jan 15;11:615669. doi: 10.3389/fmicb.2020.615669 (PMC7843509; doi:10.3389/fmicb.2020.615669)
Supplement: Supplementary file 12 [file Table_11.pdf]

Supplementary Table 11. The Average Length of Nucleotides Added and Trimmed in the Junctional Regions of IgG Repertoires

Junctional Modification	Average Length (nt)									
	3VP	5DP	3DP	5JP	N1	N2	3VT	5DT	3DT	5JT
**HH**	1.45±0.69	1.59±0.84	1.27±0.55	1.40±0.83	7.42±5.52	7.37±5.76	2.55 ±1.84	7.74±5.17	6.62±4.19	7.49±5.34
**IHB**	1.53±0.78	1.52±0.70	1.18±0.53	1.30 ±0.59	7.82±5.62	7.08±5.33	2.73 ±1.92	7.72±5.18	6.34 ±4.35	7.92±5.46
**CHB**	1.48±0.70	1.55 ±0.64	1.31 ±0.58	1.33±0.63	7.81 ±5.46	7.55±5.93	2.53 ±1.92	7.91±5.63	6.37 ±4.29	7.92 ±5.52
Cohen's d [a] **(HH *vs.* IHB)**	0.15	0.12	0.29	0.19	0.01	0.009	0.05	0.005	0.02	0.01
Cohen's d [a] **(HH *vs.* CHB)**	0.08	0.07	0.14	0.13	0.01	0.005	0.006	0.06	0.01	0.01
Cohen's d [a] **(IHB *vs.* CHB)**	0.08	0.08	0.41	0.07	0.0005	0.01	0.06	0.06	0.002	0.00003

a: Calculated by Student's t test; *Cohen's d* Value: when d ≥ 0.20 , the difference were considered to be significant.
